# Nrf2 Activation Protects Mouse Beta Cells from Glucolipotoxicity by Restoring Mitochondrial Function and Physiological Redox Balance

**DOI:** 10.1155/2019/7518510

**Published:** 2019-11-11

**Authors:** Johanna Schultheis, Dirk Beckmann, Dennis Mulac, Lena Müller, Melanie Esselen, Martina Düfer

**Affiliations:** ^1^University of Münster, Pharmaceutical and Medicinal Chemistry, Dept. of Pharmacology, Corrensstraße 48, 48149 Münster, Germany; ^2^University of Münster, Pharmaceutical Technology and Biopharmacy, Corrensstraße 48, 48149 Münster, Germany; ^3^University of Münster, Institute of Food Chemistry, Corrensstraße 45, 48149 Münster, Germany

## Abstract

Influencing the redox balance of pancreatic beta cells could be a promising strategy for the treatment of diabetes. Nuclear factor erythroid 2p45-related factor 2 (Nrf2) is present in beta cells and regulates numerous genes involved in antioxidant defense. As reactive oxygen species (ROS) are important for beta cell signaling but induce oxidative stress when present in excess, this study elucidates the influence of Nrf2-activating compounds on different kinds of ROS and correlates changes in redox balance to effects on mitochondrial function, insulin release, and cell viability. Acute glucose stimulation (15 mmol/L) of murine islet cells of C57Bl/6N mice affects ROS and redox status of the cells differently. Those ROS monitored by dihydroethidium, which detects superoxide radical anions, decrease. By contrast, oxidant status, monitored by dichlorodihydrofluorescein, as well as intracellular H_2_O_2_, increases. Glucolipotoxicity completely prevents these fast, glucose-mediated alterations and inhibits glucose-induced NAD(P)H production, mitochondrial hyperpolarization, and ATP synthesis. Oltipraz (10 *μ*mol/L) or dimethyl fumarate (DMF, 50 *μ*mol/L) leads to nuclear accumulation of Nrf2, restores mitochondrial activity and glucose-dependent ROS turnover, and antagonizes glucolipotoxicity-induced inhibition of insulin release and apoptosis. Importantly, these beneficial effects only occur when beta cells are challenged and damaged by high lipid and carbohydrate supply. At physiological conditions, insulin release is markedly reduced in response to both Nrf2 activators. This is not associated with severe impairment of glucose-induced mitochondrial hyperpolarization or a rise in apoptosis but coincides with altered ROS handling. In conclusion, Nrf2 activators protect beta cells against glucolipotoxicity by preserving mitochondrial function and redox balance. As our data show that this maintains glucose-stimulated insulin secretion, targeting Nrf2 might be suited to ameliorate progression of type 2 diabetes mellitus. By contrast, nonstressed beta cells do not benefit from Nrf2 activation, thus underlining the importance of physiological shifts in ROS homeostasis for the regulation of beta cell function.

## 1. Introduction

In early stages of type 2 diabetes mellitus, pancreatic beta cells try to compensate insulin resistance of fat, muscle, and liver by elevated hormone secretion. Thus, impaired glucose tolerance is accompanied by hyperinsulinemia. With the progression of diabetes, beta cells are severely damaged and finally fail to meet the increased demand. Consequently, therapeutic strategies making beta cells more resistant to cellular stress induced by continuing high carbohydrate and/or lipid intake, termed as glucolipotoxicity, are urgently needed. Since the discovery of the comparatively low level of antioxidant enzymes in pancreatic beta cells in the late 1990s [1], mechanisms to reinforce antioxidant defense pathways are objects of research. As activating individual pathways, e.g., solely catalase or superoxide dismutase, has turned out to be less effective [2, 3], targeting antioxidant signaling mechanisms in a more general way may be more appropriate. Nuclear factor erythroid 2p45-related factor 2 (Nrf2) is a transcription factor that regulates the expression of several genes involved in redox metabolism [4–6]. Nuclear translocation of Nrf2 requires dissociation of its adaptor protein Keap1 in the cytosol. This is achieved by oxidants and electrophiles, e.g., via formation of covalent bonds with different cysteine residues of Keap1. Known Nrf2-activating compounds are dimethyl fumarate (DMF), oltipraz, sulforaphane, tert-butylhydroquinone, and dihydro-CDDO-trifluorethyl-amide.

Human and animal studies revealed that diabetes mellitus variably influences Nrf2-dependent pathways in different cell types: In renal biopsies of patients with diabetic nephropathy, elevation of oxidative stress markers coincided with nuclear accumulation of Nrf2 [7]. In retinal tissue of humans with diabetic retinopathy, Nrf2 mRNA and protein were elevated, but activation of target genes was reduced [8]. Nrf2 and its downstream signaling molecules were downregulated in the skin tissue of patients with diabetes [9] and in fibroblasts of diabetic rats exposed to oxidative stress [10]. Insulin resistance coincided with elevated levels of Keap1 and reduced amounts of antioxidant enzymes in adipose tissue in a high-fat diet mouse model [11]. Experiments with Zucker diabetic fatty rats showed that Nrf2 staining in beta cells rises soon after the start of a high-fat diet. While beta cell damage, apparent as a reduction in insulin-positive islet area, was mostly reversible after a short period of a high-fat diet, regenerative capacity disappeared after prolonged intake [12]. Altogether, this points not only to a tissue- and organ-dependent activation of Nrf2 but also to impairment of Nrf2 signaling in response to diabetes or malnutrition. Consequently, amongst other parameters involved in balancing oxidative stress, Nrf2 was suggested to serve as a biomarker to assess the risk profile for cardiovascular disease [13].

Insight into the therapeutic potential of Nrf2 for antidiabetic therapy is derived from experiments with animal models. Db/db mice with genetic upregulation of Nrf2 activity via Keap1 knockdown were protected against the loss of glycemic control [14]. In line with this observation, treatment of mice on a high-fat diet with synthetic or natural Nrf2 activators reduced food intake and obesity [15] and ameliorated insulin resistance [11, 16]. Protective effects were mainly ascribed to reduced lipogenesis and decreased glucose disposal [11, 15], but Nrf2 can also influence the endocrine pancreas (reviewed in [17]). It was demonstrated that Nrf2-activating compounds increased the expression of antioxidant target genes [18–21]. Furthermore, a positive influence of Nrf2 activators on insulin secretion in response to an oral glucose tolerance test was reported for diabetic db/db mice. This effect was accompanied by an elevation of an insulin-positive area in pancreatic sections [14]. Therefore, it was suggested that activators of Nrf2 could be used to counteract beta cell damage induced by overnutrition or inflammation [22]. So far, the *in vivo* data and subsequent analysis of islet histology provide evidence for the protection of the endocrine pancreas by Nrf2 activators applied during the development of type 2 diabetes mellitus. Of note, studies addressing beta cells and Nrf2 activators *ex vivo* are mainly limited to stress models with H_2_O_2_ and focus on the importance of Nrf2-regulated genes for beta cell death [18, 19, 21]. The direct influence of Nrf2-activating compounds on functional parameters, such as ATP production or insulin release in response to the pathophysiologically relevant challenge of beta cells by high glucose and lipid concentrations, remains to be elucidated.

Although pancreatic islets are susceptible to oxidative stress, reactive oxygen species (ROS) are not harmful *per se* but can serve as important signaling molecules in beta cells, if concentrations are not too high [23, 24]. Consequently, strategies targeting antioxidant capacity have to be analyzed carefully. Up to now, the effects of permanently elevated glucose and lipid concentrations on physiologically generated ROS (e.g., via mitochondrial metabolism) during acute stimulation of beta cells by nutrients have not been investigated in detail. Furthermore, the impact of Nrf2 on (patho)physiological changes in ROS in pancreatic islets is not known. The present study elucidates the changes in different kinds of ROS induced by glucolipotoxic cell stress in correlation with reduction equivalents, mitochondrial function, apoptosis, and insulin release. The susceptibility of these parameters to Nrf2-activating compounds was characterized in response to high glucose/lipid load as well as under standard conditions.

## 2. Material and Methods

### 2.1. Cell and Islet Preparation

Experiments were performed with islets of Langerhans from adult C57Bl/6N mice (Charles River, Sulzfeld, Germany). The principles of laboratory animal care were followed according to German laws. Mice were euthanized using CO_2_. Islets were isolated by collagenase digestion and cultured in RPMI 1640 medium (11.1 mmol/L glucose) supplemented with 10% fetal calf serum, 100 U/mL penicillin, and 100 *μ*g/mL streptomycin at 37°C in a 5% CO_2_ humidified atmosphere. After preparation, islets or dispersed islet cells were kept overnight in standard culture medium. In experiments with oltipraz or DMF, Nrf2 activators were added during the overnight culture period (12-16 h). Next day, medium was replaced by glucolipotoxic medium (25 mmol/L glucose and 100 *μ*mol/L palmitate) or the respective control medium for 30 min up to 48 h as indicated in the figures (Nrf2 activators still present). For the investigation of apoptosis induced by T0901317 (10 *μ*mol/L), glucose concentration amounted to 33 mmol/L and islet cells were cultured in this medium with or without oltipraz for 48 h or seven days.

### 2.2. Solutions and Chemicals

Insulin secretion was determined in a bath solution of (mmol/L) 122 NaCl, 4.7 KCl, 1.1 MgCl_2_, 2.5 CaCl_2_, and 10 HEPES (pH 7.4). All other parameters were measured at 37°C in a solution containing (mmol/L) 140 NaCl, 5 KCl, 1.2 MgCl_2_, 2.5 CaCl_2_, and 10 HEPES (pH 7.4). Glucose was added as indicated. For glucolipotoxic medium, palmitate was dissolved in 0.1 N NaOH and further diluted in double-distilled water containing 0.56% fat-free bovine serum albumin. This solution was added to the culture medium in a proportion of 1 : 10 to obtain a final palmitate concentration of 100 *μ*mol/L. Control medium contained the respective amount of fat-free bovine serum albumin.

Collagenase P was obtained from Roche Diagnostics (Mannheim, Germany), and RPMI 1640, fetal calf serum, and penicillin/streptomycin were obtained from Life Technologies (Darmstadt, Germany). Rat insulin and dihydroethidium were ordered from Biotrend (Köln, Germany), and BES-H_2_O_2_ was from Wako Chemicals (Neuss, Germany). Protease inhibitor cocktail was from Roth (Karlsruhe, Germany). Antibodies were obtained from Abcam (Cambridge, UK) or Santa Cruz (Heidelberg, Germany). All other chemicals were purchased from Sigma-Aldrich (Taufkirchen, Germany) or Diagonal (Münster, Germany).

### 2.3. Insulin Secretion

After culture of the islets for 2 or 48 h under control or glucolipotoxic conditions with or without Nrf2-activating drugs, islets were silenced by incubation in bath solution with 5.6 (2 h) and 3 (1 h) mmol/L glucose. Nrf2 activators were not present in the 1 h period and in the following secretion experiment. For the determination of insulin release, batches of five islets were incubated at 37°C for 60 min with the indicated glucose concentrations. Secretion was stopped by fast cooling, and insulin concentration was quantified by radioimmunoassay using rat insulin as the standard.

### 2.4. Determination of ROS and Redox Status

Glucose-induced alterations in redox signaling in response to acute stimulation were determined by incubating islet cells in bath solution with 0.5, 3, or 15 mmol/L glucose at 37°C for 1 h. 2′,7′-Dichlorodihydrofluorescein-diacetate (DCDHF-DA, 20 *μ*mol/L), 3′-O-acetyl-6′-O-pentafluorobenzenesulfonyl-2′-7′-difluorofluorescein-acetate (BES-H_2_O_2_-Ac, 5 *μ*mol/L), or dihydroethidium (DHE, 1 *μ*mol/L) was added for the last 15 min of the 1 h period.

To detect the accumulation of H_2_O_2_, superoxide anion radicals, or altered redox status in response to different culture conditions, medium was removed after the respective culture period and cells were loaded with fluorescence dye in bath solution with 15 mmol/L glucose at 37°C for 15 min (DCDHF, DHE) or with 10 mmol/L glucose for 60 min (BES-H_2_O_2_). Fluorescence was excited at 480 nm, and emission was measured by a digital camera (filter 515 nm).

### 2.5. Determination of Mitochondrial Membrane Potential (*ΔΨ*_m_) and NAD(P)H

For the determination of Δ*Ψ*_m_, cells were incubated in bath solution with 0.5 or 15 mmol/L glucose for 1 h and loaded with rhodamine 123 (26 *μ*mol/L, 37°C) in the last 15 min. Fluorescence was normalized to maximal depolarization obtained with NaN_3_ (10 mmol/L) in each experiment. A lower percentage value indicates a more hyperpolarized status. To detect NAD(P)H autofluorescence, cells were incubated in bath solution with 0.5 mmol/L glucose for 1 h. Changes in NAD(P)H were determined after stimulation with 15 mmol/L glucose (compared to 0.5 mmol/L glucose). Excitation was achieved at 480 nm (*ΔΨ*_m_) or 360 nm (NAD(P)H), and emission was measured as described above.

### 2.6. Determination of ATP Content

After being cultured under control or glucolipotoxic conditions in the presence or absence of oltipraz for 48 h, islets were silenced in bath solution containing 6 mmol/L glucose for 1 h. Subsequently, 20 islets per batch were incubated in bath solution with either 0.5 or 15 mmol/L glucose at 37°C for 30 min. Islets were lysed and incubated at 60°C to inactivate enzymes for 20 min. ATP content was measured using a luciferin/luciferase-based assay according to the manufacturer's protocol (ATP determination kit, Invitrogen™, Thermo Fisher Scientific).

### 2.7. Apoptotic Cell Death

According to the manufacturer's protocol (in situ cell death detection kit, fluorescein, Roche Diagnostics), cells were washed with phosphate-buffered saline and fixed with 3% paraformaldehyde. Cells were permeabilized on ice for 2 min (0.1% Triton-X) and washed again. Each sample was treated with TUNEL reaction mixture and incubated in a humidified atmosphere for 1 h. Thereafter, cells were washed, nuclei were stained with bisbenzimide (Hoechst-33258), and fluorescence of apoptotic cells was excited at 480 nm. Nuclear staining was excited at 380 nm.

### 2.8. Nrf2 Immunofluorescence Staining

After treatment as indicated, cells were fixed in paraformaldehyde (3%, 1 h, room temperature) and permeabilized by Triton-X/sodium dodecyl sulfate (0.25%/1%, 10 min). To reduce nonspecific binding, normal goat serum blocking solution was used (30 min). Cells were incubated with anti-Nrf2 primary antibody (Abcam, ab62352, 1 : 200) in the dark at 37°C for 10 h. Thereafter, cells were washed 5 times with phosphate buffer. Alexa Fluor 488®-coupled secondary antibody was added (ab150077, 1 : 1000) for 1 h. Finally, cells were embedded in mounting medium containing 4′,6-diamidine-2′-phenylindole (Fluoroshield® with DAPI).

### 2.9. Western Blot Analysis

Approximately 400 islets per condition were lysed with RIPA buffer (mmol/L): 65 TRIS, 150 NaCl, 0.9 EDTA, and 1% Nonidet-P40 (10%), containing 1% protease inhibitor cocktail (freshly added) and 0.1% dithiothreitol. Samples were homogenized by an ultrasonic homogenizer (SonoPuls GM mini20, BANDELIN) for 30 s, and protein content was determined by a Bradford assay. Samples were diluted to a standardized protein concentration. Proteins were separated on a 10% polyacrylamide gel and blotted on a nitrocellulose membrane (Amersham™ Protan®, VWR, Germany), followed by incubation with anti-Nrf2 primary antibody (Abcam, ab62352, 1 : 1000) at 4°C up to 48 h. PCNA protein was used as the loading control (Santa Cruz, sc-25280 1 : 500). The secondary antibody (Cell Signaling Technology, anti-rabbit, No. 7074 1 : 1000) was added at room temperature for 1 h. All antibody dilutions were done with buffer containing 5% nonfat dry milk. Chemiluminescence (WesternBright™ Sirius™, Advansta Inc.) was detected with CemiDoc™ XRS (Bio-Rad).

### 2.10. Data Evaluation and Statistical Analysis

Data were collected from islets or islet cells of at least three independent mouse preparations for each series of experiments. Values are given as the means ± SEM. For the evaluation of *ΔΨ*_m_, six consecutive data points (10 s intervals) directly before the addition of NaN_3_ were averaged and normalized to maximal depolarization. For the analysis of NAD(P)H, all those cells displaying a reversible reaction in response to 15 mmol/L glucose were included and the difference in response to 15 *vs.* 0.5 mmol/L glucose was calculated (6 consecutive data points, 3 s intervals). Apoptosis was determined by counting the number of TUNEL-positive cells in relation to all cells in 10 randomly selected fields of each sample. Confocal images were taken by an iMIC digital microscope 2.0 (FEI, Munich, Germany) or with a IX81 fluorescence microscope (Olympus, Hamburg, Germany) with the following filter systems (DAPI/Alexa Fluor 488®): excitation at 360-370 nm/460-500 nm, dichroic mirror at 400 nm/505 nm, and emission at 426-446 nm/510-560 nm. Images were taken as multilayer stacks with a minimum of 12 images. Out of focus, fluorescence was reduced by deconvolution (Wiener filter, cellSens Dimension Software 1.17). Western blot band intensities were analyzed with Image Lab 5.0 Software (Bio-Rad). Statistical significance was assessed by Student's *t*-test or by ANOVA followed by Student-Newman-Keuls *post hoc* test for multiple comparisons. Values of *p* ≤ 0.05 were considered significant.

## 3. Results

### 3.1. Glucolipotoxicity Reduces Insulin Secretion and Influences Acute Effects of Glucose on Redox Homeostasis

**Figure 1:**
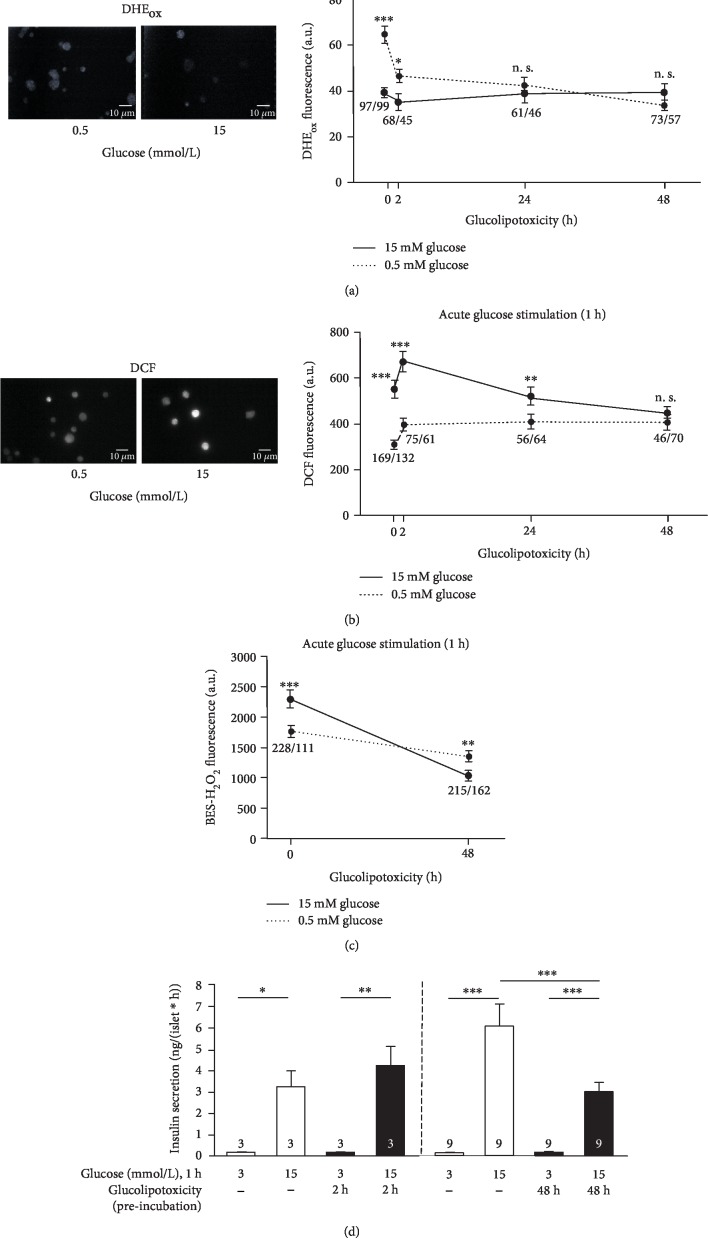
Glucolipotoxicity impairs insulin release and alters acute, glucose-induced changes in redox balance. (a–c) Mouse islet cells were cultured in control medium (10 mmol/L glucose) or in glucolipotoxic culture medium (100 *μ*mol/L palmitate and 25 mmol/L glucose) for up to 48 h. Thereafter, they were stimulated with bath solution supplemented with either 0.5 or 15 mmol/L glucose for 1 h and changes in fluorescence of DHE_ox_ ((a), indicative of superoxide anion formation), DCF ((b), indicative of a more oxidized redox status), and BES-H_2_O_2_ ((c), indicative of H_2_O_2_ accumulation) were determined. (d) Insulin release of murine islets was measured in response to 3 and 15 mmol/L glucose (1 h steady-state incubation) after 2 or 48 h in culture medium with 100 *μ*mol/L palmitate and 25 mmol/L glucose *vs.* control (10 mmol/L glucose). In (a) and (b), representative images are shown on the left. Numbers in the graph indicate the number of islet cells ((a–c): 0,5/15 mmol/L glucose) or independent preparations (d). ^∗^*p* ≤ 0.05, ^∗∗^*p* ≤ 0.01, and ^∗∗∗^*p* ≤ 0.001; 15 *vs.* 0.5 or 3 mmol/L glucose, n. s.: not significant.

Redox status and ROS play a crucial role in beta cell physiology and in the process of beta cell exhaustion by excessive nutrient supply. Acute stimulation of murine beta cells by 15 mmol/L glucose for 1 h induced alterations in cellular redox balance compared to beta cells treated with 0.5 mmol/L glucose for 1 h. ROS determined by DHE oxidation to ethidium and 2-hydroxyethidium (summarized as “DHE_ox_”) in the presence of the stimulatory glucose concentration were lower compared to the substimulatory glucose concentration (Figure 1(a) point “0”, continuous *vs.* dotted line). Amongst others, this indicates a decrease in accumulation of superoxide radical anions. By contrast, oxidation of DCDHF to 2′,7′-dichlorofluorescein (DCF) increased in response to a 1-hour stimulation with 15 mmol/L *vs.* 0.5 mmol/L glucose (Figure 1(b) point “0”, continuous *vs.* dotted line). With 3 mmol/L glucose, which is the substimulatory concentration routinely used to determine basal insulin secretion, the degree of DHE_ox_ and DCF was similar to 0.5 mmol/L glucose (Suppl. Fig. [Supplementary-material supplementary-material-1]). To test whether acute glucose stimulation leads to H_2_O_2_ accumulation, the fluorescence dye BES-H_2_O_2_ [25] was used.  Figure 1(c) (point “0”) illustrates that fluorescence is higher in response to 1 h stimulation with 15 mmol/L compared to 0.5 mmol/L glucose. The decrease in the fluorescence of the oxidation products of DHE with concomitant increase in BES-H_2_O_2_ fluorescence might indicate elevated formation of H_2_O_2_ by dismutation of superoxide radical anions.

H_2_O_2_ was suggested to contribute to acute, physiological regulation of insulin secretion [24]. To test for any influence of glucolipotoxicity on the physiological glucose-induced shifts in redox homeostasis described above, changes in redox balance and insulin release were determined after short- and long-term culture in medium supplemented with 25 mmol/L glucose and 100 *μ*mol/L palmitate. Glucolipotoxic conditions were without any negative effect on insulin release after a short period of 2 h but, as expected, significantly reduced glucose-stimulated insulin release after 48 h (Figure 1(d)). Correspondingly, the difference in DCF or DHE_ox_ fluorescence in response to the two glucose concentrations declined with increased duration of glucolipotoxic culture. It completely disappeared when cells were stimulated with 0.5 or 15 mmol/L glucose subsequent to the 48 h culture under glucolipotoxic conditions (Figures 1(a) and 1(b), point “48 h”). With respect to fluorescence of BES-H_2_O_2_, glucose-mediated changes even reversed, i.e., acute stimulation with 15 *vs.* 0.5 mmol/L glucose, resulted in a significantly reduced level instead of an increased level of H_2_O_2_ after glucolipotoxic culture for 48 h (Figure 1(c)).

### 3.2. Long-Term Treatment with Glucolipotoxicity Shifts the Redox Status in Beta Cells

**Figure 2:**
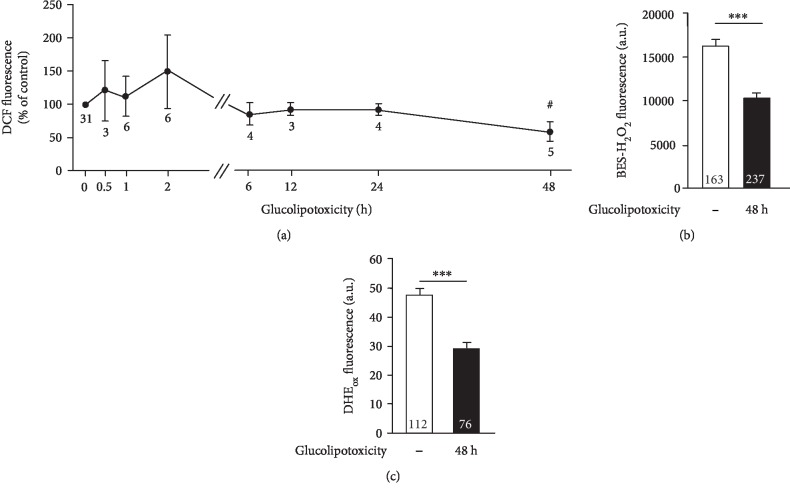
Culturing beta cells under glucolipotoxic conditions decreases the intracellular level of oxidation. Mouse islet cells were incubated in medium supplemented with 100 *μ*mol/L palmitate and 25 mmol/L glucose *vs.* standard condition (10 mmol/L glucose) for different time periods up to 48 h. Thereafter, fluorescence of DCF was determined. (b, c) The same protocol as in (a) but with BES-H_2_O_2_ or DHE as fluorescent dyes. Numbers in the graph denote the number of independent preparations (a), and numbers in bars indicate the number of islet cells (b, c). ^#^*p* ≤ 0.05*vs.* standard condition (time point “0”); ^∗∗∗^*p* ≤ 0.001.

To investigate the influence of glucolipotoxicity on intracellular ROS accumulation *per se*, beta cells were cultured as described above. Thereafter, DCF, BES-H_2_O_2_, and DHE_ox_ fluorescence were determined without any further acute treatment with 0.5 or 15 mmol/L glucose. Compared to control conditions, all indicators showed reduced fluorescence in response to the 48 h culture period in glucolipotoxic medium (Figures 2(a)–2(c)). This unexpected shift in redox balance to a less oxidized status might be a consequence of activation of antioxidant defense pathways during the 48 h culture period. The kinetics of changes in DCF fluorescence illustrated in Figure 2(a) show a tendency to increased DCF fluorescence during the first 2 h in glucolipotoxic medium, but this did not reach statistical significance. After 48 h, DCF fluorescence was decreased compared to the starting point (absolute values, control: 1557 ± 198 a.u., *n* = 144 cells, *vs.* 48 h glucolipotoxicity: 688 ± 43 a.u., *n* = 150 cells, *p* ≤ 0.001). To exclude that these results are restricted to one special culture condition, another model for glucolipotoxicity was used. In this setup, the liver X receptor agonist T0901317 (10 *μ*mol/L), which activates cellular lipid synthesis, was combined with 33 mmol/L glucose. In agreement with our other results, the treatment of beta cells with these glucolipotoxic conditions for 48 h also resulted in a less oxidized redox status (DCF fluorescence: decreased to 78.5 ± 3.9% of the initial value, *n* =  3 independent preparations, *p* ≤ 0.05; DHE_ox_ fluorescence: decrease from 40 ± 2 to 29 ± 2 a.u., *n* = 89 and 92 cells, respectively, *p* ≤ 0.001).

### 3.3. Influence of Nrf2 Activation in Nonstressed Beta Cells

**Figure 3:**
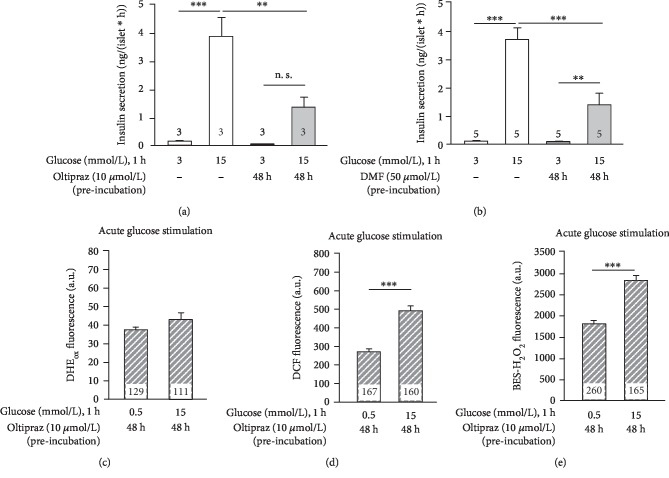
At physiological conditions, activation of Nrf2 inhibits insulin secretion and changes the glucose-dependent fast alterations in cellular ROS. (a, b) Murine islets were cultured in standard medium in the absence (white bars) or presence (grey bars) of oltipraz (10 *μ*mol/L) or DMF (50 *μ*mol/L) for 48 h. Thereafter, islets were stimulated with 3 or 15 mmol/L glucose for 1 h and insulin release was measured. (c–e) The same procedure as described for (a, b) but with dispersed islet cells. Acute 1 h stimulation with 15 mmol/L glucose did not reduce DHE-sensitive ROS compared to 0.5 mmol/L glucose (c) but still elevated DCF and BES-H_2_O_2_ fluorescence (d, e). Numbers in bars indicate the number of independent preparations (a, b) or cells (c–e). ^∗∗^*p* ≤ 0.01 and ^∗∗∗^*p* ≤ 0.001; n. s.: not significant.

Nrf2 is known as an important regulator for antioxidant defense pathways. To unravel whether activation of Nrf2 protects against glucolipotoxicity by restoring the physiological glucose-mediated changes in ROS balance, two Nrf2 activators, oltipraz and dimethyl fumarate (DMF), were used. First, the influence of both compounds on islets cultured in standard conditions was tested. 48 h culture of mouse islets with oltipraz (10 *μ*mol/L) or DMF (50 *μ*mol/L) did not affect basal insulin secretion (3 mmol/L glucose) but dramatically reduced insulin release in response to 15 mmol/L glucose to approximately 35-40% compared to controls (Figures 3(a) and 3(b)). As the influence of Nrf2 activation on insulin release under physiological conditions was clearly negative, we tested for any effects on glucose-mediated changes in beta cell ROS. Contrasting to the effects illustrated in Figure 1(a), DHE_ox_ fluorescence did not differ in response to stimulation with 15 vs. 0.5 mmol/L glucose after treatment of beta cells with oltipraz for 48 h (Figure 3(c)). The physiological rise in DCF fluorescence that was observed by acutely stimulating beta cells with 15 mmol/L compared to 0.5 mmol/L glucose was still present but lower (483 ± 22 a.u., n = 160, *vs.*567 ± 34 a.u., *n* = 132, *p* ≤ 0.01) after 48 h culture of beta cells with medium supplemented with oltipraz (Figure 3(d), compared to the point of origin of the continuous line in Figure 1(b)). This indicates a less oxidized status of the beta cells in response to glucose after treatment with oltipraz. However, with respect to H_2_O_2_, acute stimulation with 15 mmol/L glucose increased fluorescence of BES-H_2_O_2_ significantly more in beta cells pretreated with oltipraz compared to control (right bar in Figure 3(e)*vs.* point of origin of the continuous line in Figure 1(c): 2781 ± 100 a.u., *n* = 165, *vs.*2291 ± 128 a.u., *n* = 111, *p* ≤ 0.001).

### 3.4. Restoration of the Glucose-Dependent Changes in Redox Balance by Nrf2 Activation during Glucolipotoxic Culture

**Figure 4:**
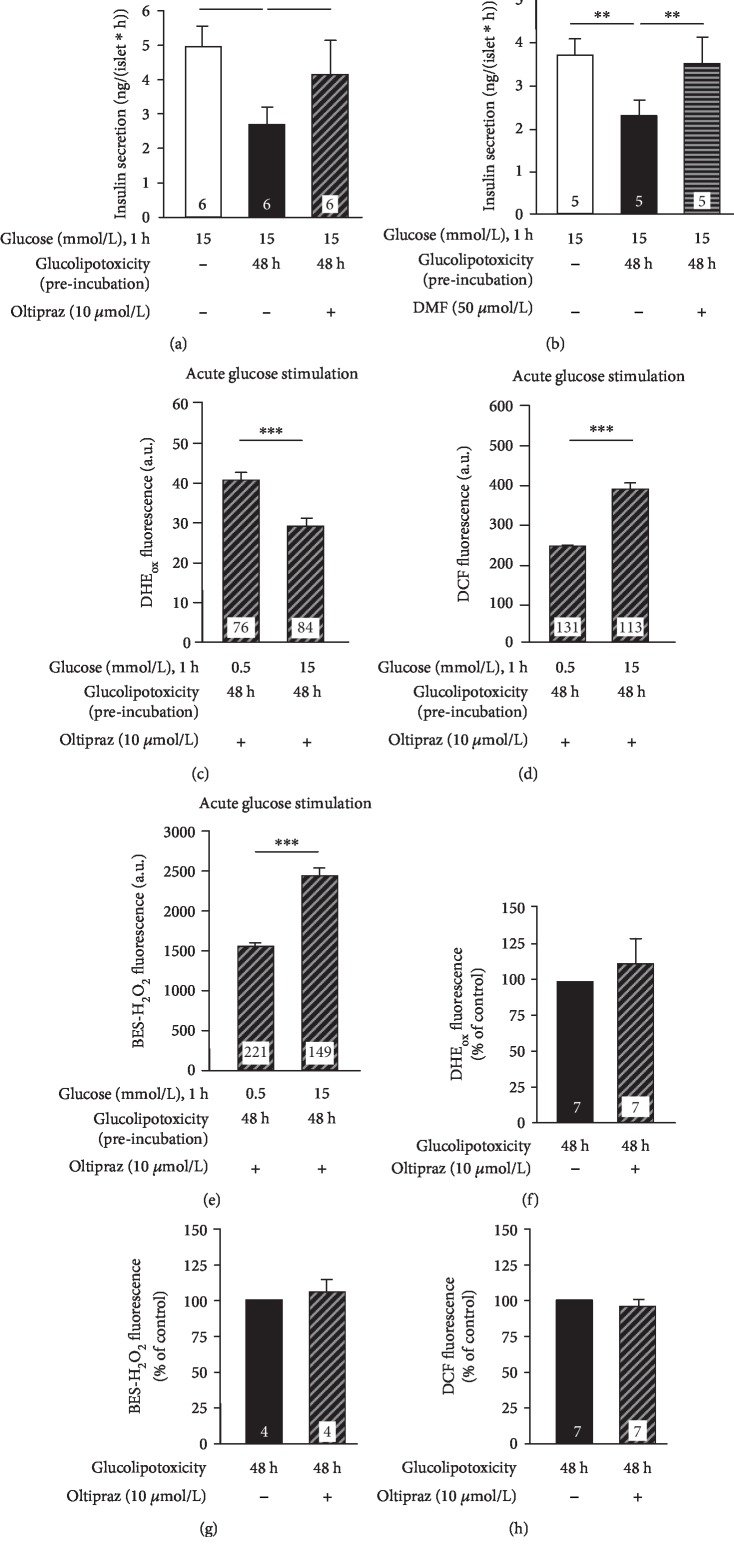
Activation of Nrf2 protects the physiological redox balance and insulin secretion from glucolipotoxic damage. Oltipraz (10 *μ*mol/L) or DMF (50 *μ*mol/L) was added 12-16 h before changing standard medium to glucolipotoxic medium and during glucolipotoxic culture (48 h). (a, b) Oltipraz and DMF prevented the detrimental effect of glucolipotoxicity on glucose-stimulated insulin secretion (1 h steady-state incubation). (c–e) Alterations of DHE_ox_, DCF, and BES-H_2_O_2_ fluorescence in mouse islet cells in response to acute glucose stimulation (1 h) were preserved in islet cells after culture in glucolipotoxic medium when Nrf2 was activated by oltipraz. (f–h) Redox status of islet cells directly after glucolipotoxic culture was not affected by pretreatment with oltipraz. Numbers in bars indicate the number of independent preparations (a, b, and f–h) or cells (c–e). ^∗^*p* ≤ 0.05, ^∗∗^*p* ≤ 0.01, and ^∗∗∗^*p* ≤ 0.001.

The data described above showed that activation of Nrf2 under physiological conditions inhibited insulin release and altered the redox profile in response to acute glucose stimulation. Next, we checked whether activation of Nrf2 could protect against glucolipotoxic effects. In these experiments, oltipraz (10 *μ*mol/L) or DMF (50 *μ*mol/L) was added before (12-16 h) and during glucolipotoxic culture (48 h). Both Nrf2 activators protected against inhibition of insulin release by glucolipotoxicity (Figures 4(a) and 4(b)). Coincubation of islet cells with oltipraz (10 *μ*mol/L) largely restored the physiological glucose-induced shifts in the redox profile, i.e., an increase in DCF fluorescence and elevated oxidation of BES-H_2_O_2_ as well as a decrease in DHE_ox_ fluorescence in response to 1-h stimulation with 15 mmol/L *vs.* 0.5 mmol/L glucose (Figures 4(c)–4(e), compared to Figures 1(a)–1(c)). By contrast, ROS accumulation directly after 48 h of glucolipotoxicity was not affected (Figures 4(f)–4(h)).

**Figure 5:**
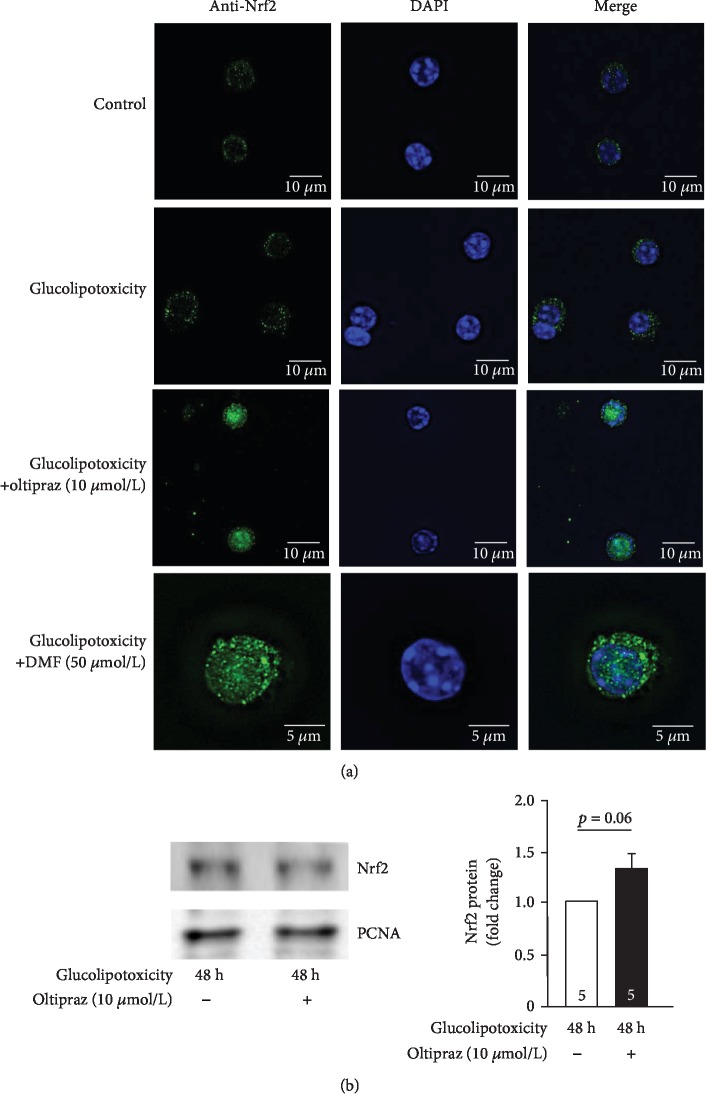
Oltipraz and DMF alter Nrf2 distribution in islet cells stressed by glucolipotoxicity. Cells were treated by Nrf2 activators and cultured in glucolipotoxic medium as described for Figure 4. (a) Representative images out of 3-5 mouse preparations are shown (left: anti-Nrf2 antibody (green), middle: DAPI staining (blue), and right: overlay). Addition of oltipraz (10 *μ*mol/L) or DMF (50 *μ*mol/L) during glucolipotoxic culture increased nuclear Nrf2 localization. (b) Islets were lysed and analyzed by Western blot after 48 h culture with or without oltipraz. Representative blots for Nrf2 protein (95 kDa) and the housekeeping protein PCNA (36 kDa) are shown. Coculture with oltipraz did not significantly elevate Nrf2 protein.

Alterations in intracellular Nrf2 localization were monitored under the same conditions. After control or glucolipotoxic culture for 48 h, Nrf2 staining was weak and mainly cytosolic, appearing diffused or dot-like (Figure 5(a), upper traces). Nuclear Nrf2 staining clearly increased when oltipraz or DMF was present during glucolipotoxic culture (Figure 5(a), lower traces). Western blot analysis was performed to elucidate whether Nrf2 protein expression was increased by Nrf2 activating compounds. The data presented in Figure 5(b) illustrate that Nrf2 protein tends to increase in islets exposed to glucolipotoxic medium and oltipraz but the effect was not statistically different.

### 3.5. Protection against Impairment of Mitochondrial Function by Nrf2 Activation

**Figure 6:**
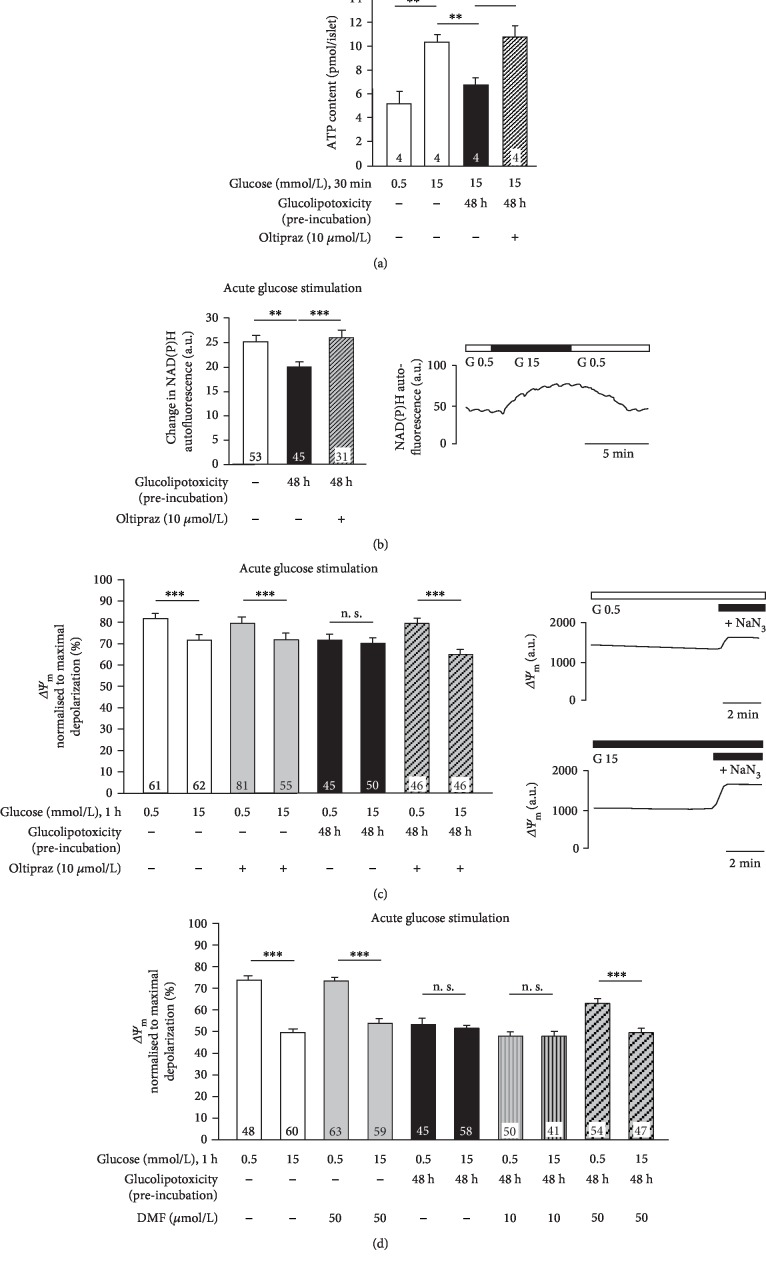
Activation of Nrf2 prevents the inhibitory effects of glucolipotoxicity on ATP content, glucose-induced generation of NAD(P)H, and mitochondrial function. Oltipraz (10 *μ*mol/L) or DMF (10 or 50 *μ*mol/L) was added to isolated murine islet cells 12-16 h before changing standard medium to glucolipotoxic medium and during glucolipotoxic culture (48 h). (a) Glucose-stimulated rise in ATP content was decreased after glucolipotoxic culture. Oltipraz protected against this. (b) Glucose-stimulated elevation of NAD(P)H autofluorescence was reduced after glucolipotoxic culture. This was completely prevented by oltipraz. A representative recording after culture in standard medium is shown on the right. (c, d) Mitochondrial membrane potential (*ΔΨ*_m_) was determined in response to 1 h stimulation with 0.5 *vs.* 15 mmol/L glucose after culture in control (white and grey bars) or glucolipotoxic (black and hatched bars) medium in the presence or absence of Nrf2 activators. Mitochondria damaged by glucolipotoxicity were unresponsive to glucose (black bars). Oltipraz or DMF protected against this (hatched bars). In (c), representative recordings for control conditions are shown on the right. Numbers in bars indicate the number of independent preparations (a) or cells (b–d). ^∗∗^*p* ≤ 0.01 and ^∗∗∗^*p* ≤ 0.001; n. s.: not significant.

Our data show that glucolipotoxicity prevents the physiological alterations of ROS induced by acute stimulation with glucose. Activation of Nrf2 by oltipraz protects against this. In general, intracellular ROS can be produced by various mechanisms, e.g., via NADPH-dependent oxidases or mitochondrial respiration [26, 27]. As mitochondrial metabolism is an important source for superoxide radical and subsequent H_2_O_2_ generation in response to acute glucose stimulation in pancreatic beta cells, it was investigated if restoration of the physiological redox profile by oltipraz results from protective effects on mitochondrial function. To test for this, ATP content, NAD(P)H autofluorescence, and mitochondrial membrane potential were determined after glucolipotoxic cell culture (48 h). ATP content increases in response to 15 mmol/L vs. 0.5 mmol/L glucose. Glucose-mediated elevation of ATP production was markedly decreased after glucolipotoxic culture. Islets were protected by coculture with oltipraz (Figure 6(a)). In agreement with impaired ATP synthesis, the increase in NAD(P)H autofluorescence (Figure 6(b), exemplary trace) induced by elevating glucose concentration from 0.5 to 15 mmol/L was distinctly reduced after glucolipotoxic culture (Figure 6(b), black bar *vs.* white bar). This was prevented by coincubation with oltipraz (Figure 6(b), hatched bar). Stimulating beta cells with 15 mmol/L *vs.* 0.5 mmol/L glucose hyperpolarized beta cell mitochondria (Figure 6(c), white bars and exemplary traces). This effect was completely absent after glucolipotoxic culture (Figure 6(c), black bars). Coincubation of glucolipotoxicity-treated beta cells with oltipraz restored mitochondrial reactivity in response to acute glucose stimulation (Figure 6(c), hatched bars). To confirm that the protective effects resulted from an interaction with Nrf2, changes in mitochondrial membrane potential were also determined with DMF. In line with our hypothesis, preincubation with DMF (50 *μ*mol/L) also protected mitochondria against glucolipotoxic damage (Figure 6(d)).

### 3.6. Influence of Nrf2 Activation on Apoptotic Cell Death

**Figure 7:**
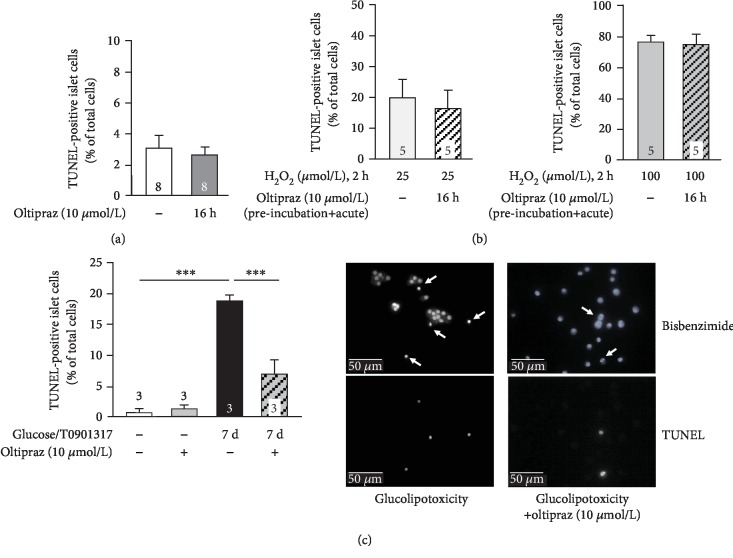
Nrf2 activation influences cell viability of islet cells. (a) Culturing mouse islet cells in standard medium with or without oltipraz (10 *μ*mol/L) for approximately 16 h did not affect the fraction of apoptotic cells. (b) After the 16 h culture period, cells were stressed by 25 or 100 *μ*mol/L H_2_O_2_ in the continued presence of oltipraz for 2 h. Apoptosis tends to be reduced by oltipraz in those cells exposed to the lower H_2_O_2_ concentration (left diagram), but not when H_2_O_2_-induced cell damage was high (right diagram). Note that scaling is different to provide adequate resolution. (c) After the 16 h culture period, medium was exchanged and culture was continued in the presence of 33 mmol/L glucose and 10 *μ*mol/L *T*0901317 in the presence or absence of oltipraz for 7 days. Nrf2 activation markedly reduced the fraction of apoptotic cells. Images of one representative experiment are shown on the right. The upper images illustrate nuclear staining. Arrows indicate TUNEL-positive cells shown in the lower traces. Numbers in bars indicate the number of independent preparations. ^∗∗∗^*p* ≤ 0.001.

As adequate release of insulin is a result of proper beta cell function and cell mass, the influence of Nrf2 activation on islet cell viability was investigated. Oltipraz (10 *μ*mol/L) did not increase the number of apoptotic islet cells under control conditions (Figure 7(a): 16 h culture; Figure 7(c): 7 d culture). This indicates that the reduced secretory response observed after culture with oltipraz under standard conditions was not mediated by elevated beta cell death. When cells were stressed with H_2_O_2_, Nrf2 activation was not able to counteract apoptosis induced by 25 or 100 *μ*mol/L H_2_O_2_ (2 h) (Figure 7(b)). Next, we elucidated the influence of glucolipotoxicity on islet cell survival. Induction of apoptosis in the palmitate/glucose model revealed a very high variability in the degree of apoptosis after 7 d (data not shown). To get a more constant response, the protocol was changed to 33 mmol/L glucose in combination with the liver X receptor agonist T0901317 (10 *μ*mol/L). After culturing islet cells under these conditions for 7 days, the fraction of apoptotic cells stably amounted to ~18%. Coculture with oltipraz did not completely prevent but significantly reduce the detrimental effect of glucolipotoxicity (Figure 7(c)).

## 4. Discussion

### 4.1. Protection against Glucolipotoxicity-Mediated Mitochondrial Impairment by Nrf2

We previously demonstrated that activation of Nrf2 by oltipraz prevented the inhibitory effect of H_2_O_2_ on insulin release [28]. Our current data show that pharmacological activation of Nrf2 also protects pancreatic beta cells from glucolipotoxicity-induced cell damage. We used a combined protocol of medium supplemented with 25 mmol/L glucose and 100 *μ*mol/L palmitate, applied for up to 48 h, in this study. This clearly supraphysiological glucose concentration was chosen as it was reported for mouse islets that 25 mmol/L glucose with or without palmitate inhibited the 1^st^ phase of insulin release after short-term incubation (3 d) [29] similar to the situation described for type 2 diabetes mellitus. Of course, one must keep in mind that *in vitro* models cannot be transferred one-to-one to the complex scenario *in vivo*. In our hands, the glucolipotoxic *in vitro* protocol reduced glucose-stimulated insulin secretion to ~40 to 50% (Figure 1(d) and Figures 4(a) and 4(b)). During this 48 h culture period, impaired islet function was not accompanied by apoptosis even in combination with 0.5 instead of 0.1 mmol/L palmitate (unpublished data). The inhibition of glucose-induced insulin release detected after glucolipotoxic culture was largely reduced in islets coincubated with oltipraz and almost prevented by DMF (Figures 4(a) and 4(b)). Our experiments reveal that this beneficial effect is mediated by preservation of mitochondrial reactivity. Mitochondria play a central role in the stimulus secretion coupling of beta cells as they couple glucose metabolism to closure of ATP-regulated K^+^ channels, membrane depolarization, and subsequent Ca^2+^ influx [30]. Prolonged exposure of beta cells to high glucose and lipid concentrations clearly reduced the ability of mitochondria to hyperpolarize in response to acute stimulation with glucose. Glucose-mediated generation of reduction equivalents and intracellular ATP was also decreased (Figure 6). These changes are indicative of an impaired activation of mitochondrial respiratory chains and are in agreement with the observations of others described for insulin-secreting cell lines as well as primary islet cells [31–33]. Activation of Nrf2 restored glucose responsiveness with respect to all metabolic parameters. Prevention of mitochondrial damage by Nrf2 has already been described for other tissues: In endothelial cells, activation of Nrf2 protected against mitochondrial depolarization and release of cytochrome c in response to high glucose concentrations. In renal tubular cells of obese and glucose intolerant db/db mice, an Nrf2-dependent pathway reduced mitochondrial fragmentation and activation of caspase 3 [34, 35].

### 4.2. Effects of Glucolipotoxicity and Nrf2 Activation on Intracellular ROS and Redox Status

While ROS have only been seen in a negative context with respect to adequate beta cell function for a long time, the role of ROS has been reinterpreted during the last decade. There is evidence that a certain amount of H_2_O_2_ supports insulin release [24], whereas dramatically increased accumulation results in severe cell damage [36]. ROS, like superoxide radical anions or hydroxyl radicals, are highly reactive and quickly oxidize membrane proteins or lipids. With respect to the fate of free radicals produced in excess, the situation is complex. Most investigations, describing an increase in ROS in response to culture with high glucose and/or lipid concentrations, were done with insulin-secreting cell lines, mainly INS-1 cells and not with primary islets [37–39]. Furthermore, many conclusions were drawn by monitoring DCDHF oxidation, which is not specific for a certain kind of ROS but rather indicates elevated oxidative reactions of different origin [40]. For primary tissue, data is limited. Our results show that those ROS, which are sensitive to the two fluorescent dyes BES-H_2_O_2_ and DHE, do not accumulate in beta cells after glucolipotoxic challenge for up to 48 h but finally even decrease. The same holds true for DCF fluorescence (Figure 2). BES-H_2_O_2_ is reported to detect H_2_O_2_ whereas DHE is preferentially oxidized by superoxide radical anion [25, 41]. In line with our results, Moore et al. observed no elevation of oxidative stress levels in rat islet cells cultured with 500 *μ*mol/L palmitate and 16.7 mmol/L glucose for 72 h [42] and Martens et al. showed a reduced concentration of superoxide radical anions in rat islet cells after culture in medium supplemented with 20 *vs.* 6 mmol/L glucose [43]. Even with a high concentration of palmitate (1 mmol/L, combined with 33 mmol/L glucose), DCF fluorescence decreased after 3 days of culture in mouse islet cells [33]. By contrast, elevation of DCF fluorescence by 400 *μ*mol/L palmitate or oleate was reported by others for rodent islets [44, 45]. The reasons for these discrepancies remain unclear, but in most cases, results cannot be directly compared due to variations in concentration, albumin binding, the nature of free fatty acid, or exposure time. Even though pancreatic islets are very sensitive to oxidative stress, it is known that they can align to such situations by modulation of antioxidant defense mechanisms [36]. An important observation of our investigation is that glucolipotoxicity prevents glucose from acutely changing intracellular redox balance irrespective of the fluorescence dye used for detection (Figures 1(a)–1(c)). Most likely, mitochondrial damage plays a major role for this loss of acute response. Our data clearly show that strengthening Nrf2-mediated pathways restores the physiological changes in ROS induced by acute glucose stimulation. Although activation of Nrf2 did not affect ROS accumulation in glucolipotoxicity-damaged beta cells *per se* (Figures 4(f)–4(h)), glucose-induced alterations of ROS, i.e., an increase in BES-H_2_O_2_ and a decrease in DHE_ox_ fluorescence in response to acute stimulation with 15 *vs.* 0.5 mmol/L glucose, reappeared when cells were treated with oltipraz (Figures 4(c)–4(e)). We hypothesize that this reflects increased flux of radicals, more precisely, the generation of superoxide radical anions as a by-product of mitochondrial metabolism during glucose stimulation and its fast dismutation to H_2_O_2_. Taken together, our data indicate that Nrf2-activating compounds protect against mitochondrial failure which is reflected by restoration of the acute shifts in ROS in response to the two different glucose concentrations. The data also show that not every change is normalized by treatment with oltipraz, but obviously, the glucolipotoxicity-induced lowering of ROS accumulation during culture is not decisive in this context.

### 4.3. Negative Effects of Nrf2 Activation

Importantly, our data reveal that Nrf2 activation is only beneficial for beta cells under pathophysiological conditions. In islets not challenged by nutrient excess, treatment with either oltipraz or DMF dramatically inhibited glucose-stimulated insulin release (Figures 3(a) and 3(b)). Remarkably, the reduction of DHE_ox_-detectable ROS in response to acute stimulation with glucose was completely prevented after Nrf2 activation and the rise in DCF fluorescence was lowered (Figures 3(c) and 3(d)). This observation is in agreement with the idea that overactivation of antioxidant capacity disturbs the physiological redox balance. In line with this hypothesis, exposure of INS-1 cells to arsenite for 96 h elevated Nrf2 levels, reduced oxidation reactions, but decreased the stimulatory effect of 20 mmol/L glucose on insulin secretion [46]. An increase in H_2_O_2_ production, induced by an acute rise in the glucose concentration, is suggested to function as part of a second messenger system that contributes to glucose-regulated insulin release, but discussion remains controversial [24, 47, 48]. Our results imply that a glucose-mediated rise in H_2_O_2_ beyond the physiological level is associated with impaired insulin release. Presumably, high concentrations of H_2_O_2_ together with accumulation of superoxide radical anions trigger beta cell dysfunction in this context. A dual role for beta cell regulation, i.e., acute, stimulatory effects at low concentrations and a negative impact at higher concentrations, is also postulated for nitric oxide [30]. The altered balance in ROS described in our study for “healthy” beta cells in response to oltipraz could be caused by subtle effects of long-lasting Nrf2 activation on mitochondrial respiratory chains. Although glucose did not lose its ability to hyperpolarize the mitochondria, the degree of mitochondrial activation tended to be smaller compared to control, when Nrf2 was activated by oltipraz or DMF (Figures 6(c) and 6(d), grey *vs.* white bars). Furthermore, temporarily elevated levels of both, H_2_O_2_ and superoxide radical anions, during acute glucose stimulation (compared to low glucose) might directly disturb signaling steps downstream to mitochondria.

### 4.4. Influence of Nrf2 Activation on Beta Cell Mass

Our experiments reveal that the proapoptotic effect of H_2_O_2_ in islet cells could not be prevented by Nrf2 activation. By contrast, oltipraz drastically reduced islet cell death induced by increased intracellular lipid synthesis and high glucose by ~65% (Figures 7(b) and 7(c)). This suggests that H_2_O_2_ is not decisive for glucolipotoxicity-mediated cell death. Partial protection against H_2_O_2_-induced cell death was described for the Nrf2 activator dh404 (dihydro-CDDO-trifluoroethyl amide) in human and rat islets [18, 19] or MIN-6 cells [21]. To our knowledge, the influence of Nrf2 on glucolipotoxicity-induced apoptosis has not yet been investigated in primary beta cells. Treatment of insulin-secreting INS-1 cells with a high glucose concentration of 25 mmol/L elevated apoptosis. Protection against this by the natural product honokiol was associated with translocation of Nrf2 to the nucleus [49]. In INS-1E cells, lipotoxic conditions (500 *μ*mol/L palmitate) transiently elevated Nrf2 protein content, thereby dampening stress-induced cell death. This effect was lost after prolonged exposure (16 h) to lipotoxicity. The underlying mechanisms are not entirely clear but seem to involve a novel pathway for regulation of Nrf2, mediated by ER-located thrombospondin [20]. The importance of Nrf2 for beta cell mass was also shown in transgenic animal models with elevated activity of iNOS. Islets of mice overexpressing iNOS displayed increased proliferation when Keap1 was knocked out concurrently compared to those without elimination of the Nrf2-inactivating protein [50].

In summary, our data demonstrate that protection of mitochondrial function is a key element of the Nrf2-mediated defense mechanism against glucolipotoxicity in beta cells. Preservation of mitochondrial activity by Nrf2 activators does not interact with the general redox status of beta cells at stress conditions but restores the physiological, fast alterations in ROS turnover, as well as insulin release in response to acute stimulation with glucose. This study emphasizes the importance of dynamic changes in redox homeostasis for the regulation of insulin release, which is severely affected by abnormally elevated glucose and lipid load. Targeting Nrf2 in patients suffering from dyslipidemia and glucose intolerance could extend current options for the treatment of metabolic disease.

## Data Availability

The data used to support the findings of this study are available from the corresponding author upon request.

## Abbreviations

a.u.: Arbitrary fluorescence units
BES-H_2_O_2_: 3′-O-acetyl-6′-O-pentafluorobenzenesulfonyl-2′-7′-difluorofluorescein
DAPI: 4′,6-Diamidine-2′-phenylindole
DCDHF-DA: 2′,7′-Dichlorodihydrofluorescein-diacetate
DCF: 2′,7′-Dichlorofluorescein
DHE: Dihydroethidium
DHE_ox_: Oxidation products of dihydroethidium
DMF: Dimethyl fumarate
iNOS: Inducible nitric oxide synthase
Nrf2: Nuclear factor erythroid 2p45-related factor 2
PCNA: Proliferating cell nuclear antigen
ROS: Reactive oxygen species.

## Supplementary Materials

Supplementary Materials Comparison of the effect of substimulatory glucose concentrations on acute changes in ROS balance. Islet cells were acutely treated with 0.5, 3, or 15 mmol/L glucose. Levels of DHE_ox_ (A) and DCF (B) fluorescence were similar with the two substimulatory glucose concentrations but change in response to 15 mmol/L glucose. Numbers in bars indicate the number of cells. ^∗∗∗^*p* ≤ 0.001.

## References

[1] Tiedge M., Lortz S., Drinkgern J., Lenzen S. (1997). Relation between antioxidant enzyme gene expression and antioxidative defense status of insulin-producing cells. *Diabetes*.

[2] Mysore T. B., Shinkel T. A., Collins J. (2005). Overexpression of glutathione peroxidase with two isoforms of superoxide dismutase protects mouse islets from oxidative injury and improves islet graft function. *Diabetes*.

[3] Lortz S., Tiedge M. (2003). Sequential inactivation of reactive oxygen species by combined overexpression of SOD isoforms and catalase in insulin-producing cells. *Free Radical Biology & Medicine*.

[4] Kim Y. C., Masutani H., Yamaguchi Y., Itoh K., Yamamoto M., Yodoi J. (2001). Hemin-induced activation of the thioredoxin gene by Nrf2. *Journal of Biological Chemistry*.

[5] Alam J., Stewart D., Touchard C., Boinapally S., Choi A. M. K., Cook J. L. (1999). Nrf2, a Cap’n’Collar transcription factor, regulates induction of the heme oxygenase-1 gene. *The Journal of Biological Chemistry*.

[6] Dreger H., Westphal K., Weller A. (2009). Nrf2-dependent upregulation of antioxidative enzymes: a novel pathway for proteasome inhibitor-mediated cardioprotection. *Cardiovascular Research*.

[7] Jiang T., Huang Z., Lin Y., Zhang Z., Fang D., Zhang D. D. (2010). The protective role of Nrf2 in streptozotocin-induced diabetic nephropathy. *Diabetes*.

[8] Zhong Q., Mishra M., Kowluru R. A. (2013). Transcription factor Nrf2-mediated antioxidant defense system in the development of diabetic retinopathy. *Investigative Ophthalmology & Visual Science*.

[9] Lee Y. J., Kwon S. B., An J. M. (2015). Increased protein oxidation and decreased expression of nuclear factor E2‐related factor 2 protein in skin tissue of patients with diabetes. *Clinical and Experimental Dermatology*.

[10] Bitar M. S., Al-Mulla F. (2011). A defect in Nrf2 signaling constitutes a mechanism for cellular stress hypersensitivity in a genetic rat model of type 2 diabetes. *American Journal of Physiology. Endocrinology and Metabolism*.

[11] Yu Z., Shao W., Chiang Y. (2011). Oltipraz upregulates the nuclear factor (erythroid-derived 2)-like 2 [corrected](NRF2) antioxidant system and prevents insulin resistance and obesity induced by a high-fat diet in C57BL/6J mice. *Diabetologia*.

[12] Abebe T., Mahadevan J., Bogachus L. (2017). Nrf2/antioxidant pathway mediates *β* cell self-repair after damage by high-fat diet–induced oxidative stress. *JCI Insight*.

[13] Robson R., Kundur A. R., Singh I. (2018). Oxidative stress biomarkers in type 2 diabetes mellitus for assessment of cardiovascular disease risk. *Diabetes and Metabolic Syndrome: Clinical Research and Reviews*.

[14] Uruno A., Furusawa Y., Yagishita Y. (2013). The Keap1-Nrf2 system prevents onset of diabetes mellitus. *Molecular and Cellular Biology*.

[15] Shin S., Wakabayashi J., Yates M. S. (2009). Role of Nrf2 in prevention of high-fat diet-induced obesity by synthetic triterpenoid CDDO-imidazolide. *European Journal of Pharmacology*.

[16] Sampath C., Rashid M. R., Sang S., Ahmedna M. (2017). Specific bioactive compounds in ginger and apple alleviate hyperglycemia in mice with high fat diet-induced obesity via Nrf2 mediated pathway. *Food Chemistry*.

[17] Pi J., Zhang Q., Fu J. (2010). ROS signaling, oxidative stress and Nrf2 in pancreatic beta-cell function. *Toxicology and Applied Pharmacology*.

[18] Masuda Y., Vaziri N. D., Li S. (2015). The effect of Nrf2 pathway activation on human pancreatic islet cells. *PLoS One*.

[19] Li W., Wu W., Song H. (2014). Targeting Nrf2 by dihydro‐CDDO‐trifluoroethyl amide enhances autophagic clearance and viability of *β*‐cells in a setting of oxidative stress. *FEBS Letters*.

[20] Cunha D. A., Cito M., Carlsson P.-O. (2016). Thrombospondin 1 protects pancreatic *β* -cells from lipotoxicity via the PERK-NRF2 pathway. *Cell Death and Differentiation*.

[21] Fu J., Zheng H., Wang H. (2015). Protective Role of Nuclear Factor E2-Related Factor 2 against Acute Oxidative Stress-Induced Pancreatic *β*-Cell Damage. *Oxidative Medicine and Cellular Longevity*.

[22] David J. A., Rifkin W. J., Rabbani P. S., Ceradini D. J. (2017). The Nrf2/Keap1/ARE pathway and oxidative stress as a therapeutic target in type II diabetes mellitus. *Journal of Diabetes Research*.

[23] Leloup C., Tourrel-Cuzin C., Magnan C. (2009). Mitochondrial reactive oxygen species are obligatory signals for glucose-induced insulin secretion. *Diabetes*.

[24] Pi J., Bai Y., Zhang Q. (2007). Reactive oxygen species as a signal in glucose-stimulated insulin secretion. *Diabetes*.

[25] Maeda H., Fukuyasu Y., Yoshida S. (2004). Fluorescent probes for hydrogen peroxide based on a non‐oxidative mechanism. *Angewandte Chemie (International Ed. in English)*.

[26] Graciano M. F., Valle M., Kowluru A., Curi R., Carpinelli A. (2011). Regulation of insulin secretion and reactive oxygen species production by free fatty acids in pancreatic islets. *Islets*.

[27] Ježek P., Dlasková A., Plecitá-Hlavatá L. (2012). Redox homeostasis in pancreatic beta cells. *Oxidative Medicine and Cellular Longevity*.

[28] Edalat A., Schulte-Mecklenbeck P., Bauer C. (2015). Mitochondrial succinate dehydrogenase is involved in stimulus-secretion coupling and endogenous ROS formation in murine beta cells. *Diabetologia*.

[29] Doliba N. M., Liu Q., Li C. (2017). Inhibition of cholinergic potentiation of insulin secretion from pancreatic islets by chronic elevation of glucose and fatty acids: protection by casein kinase 2 inhibitor. *Mol. Metab.*.

[30] Drews G., Krippeit-Drews P., Düfer M., Islam S. (2014). Electrophysiology of islet cells. *Islets of Langerhans*.

[31] Köhnke R., Mei J., Park M., York D. A., Erlanson-Albertsson C. (2007). Fatty acids and glucose in high concentration down-regulates ATP synthase beta-subunit protein expression in INS-1 cells. *Nutritional Neuroscience*.

[32] Somesh B. P., Verma M. K., Sadasivuni M. K. (2013). Chronic glucolipotoxic conditions in pancreatic islets impair insulin secretion due to dysregulated calcium dynamics, glucose responsiveness and mitochondrial activity. *BMC Cell Biology*.

[33] Barroso Oquendo M., Layer N., Wagner R., Krippeit-Drews P., Drews G. (2018). Energy depletion and not ROS formation is a crucial step of glucolipotoxicity (GLTx) in pancreatic beta cells. *Pflügers Archiv - European Journal of Physiology*.

[34] Xiao L., Xu X., Zhang F. (2017). The mitochondria-targeted antioxidant MitoQ ameliorated tubular injury mediated by mitophagy in diabetic kidney disease via Nrf2/PINK1. *Redox Biology*.

[35] Zhong Z.-Y., Tang Y. (2016). Upregulation of periostin prevents high glucose-induced mitochondrial apoptosis in human umbilical vein endothelial cells via activation of Nrf2/HO-1 signaling. *Cellular Physiology and Biochemistry*.

[36] Drews G., Krippeit-Drews P., Düfer M. (2010). Oxidative stress and beta-cell dysfunction. *Pflügers Archiv - European Journal of Physiology*.

[37] Maris M., Robert S., Waelkens E. (2013). Role of the saturated nonesterified fatty acid palmitate in beta cell dysfunction. *Journal of Proteome Research*.

[38] Ryu G. R., Yoo J. M., Lee E., Ko S.-H., Ahn Y.-B., Song K.-H. (2011). Decreased expression and induced nucleocytoplasmic translocation of pancreatic and duodenal homeobox 1 in INS-1 cells exposed to high glucose and palmitate. *Diabetes and Metabolism Journal*.

[39] Quan X., Zhang L., Li Y., Liang C. (2014). TCF2 attenuates FFA-induced damage in islet *β*-cells by regulating production of insulin and ROS. *International Journal of Molecular Sciences*.

[40] Kalyanaraman B., Darley-Usmar V., Davies K. J. A. (2012). Measuring reactive oxygen and nitrogen species with fluorescent probes: challenges and limitations. *Free Radical Biology & Medicine*.

[41] Zhao H., Kalivendi S., Zhang H. (2003). Superoxide reacts with hydroethidine but forms a fluorescent product that is distinctly different from ethidium: potential implications in intracellular fluorescence detection of superoxide. *Free Radical Biology & Medicine*.

[42] Moore P. C., Ugas M. A., Hagman D. K., Parazzoli S. D., Poitout V. (2004). Evidence against the involvement of oxidative stress in fatty acid inhibition of insulin secretion. *Diabetes*.

[43] Martens G. A., Cai Y., Hinke S., Stangé G., van de Casteele M., Pipeleers D. (2005). Glucose suppresses superoxide generation in metabolically responsive pancreatic beta cells. *The Journal of Biological Chemistry*.

[44] Oprescu A. I., Bikopoulos G., Naassan A. (2007). Free fatty acid-induced reduction in glucose-stimulated insulin secretion: evidence for a role of oxidative stress in vitro and in vivo. *Diabetes*.

[45] Joseph J. W., Koshkin V., Saleh M. C. (2004). Free fatty acid-induced beta-cell defects are dependent on uncoupling protein 2 expression. *The Journal of Biological Chemistry*.

[46] Fu J., Woods C. G., Yehuda-Shnaidman E. (2010). Low-level arsenic impairs glucose-stimulated insulin secretion in pancreatic beta cells: involvement of cellular adaptive response to oxidative stress. *Environmental Health Perspectives*.

[47] Deglasse J. P., Roma L. P., Pastor-Flores D., Gilon P., Dick T. P., Jonas J. C. (2018). Glucose acutely reduces cytosolic and mitochondrial H_2_O_2_ in rat pancreatic beta cells. *Antioxidants & Redox Signaling*.

[48] Neal A., Rountree A., Kernan K. (2016). Real-time imaging of intracellular hydrogen peroxide in pancreatic islets. *The Biochemical Journal*.

[49] Li C.-G., Ni C.-L., Yang M. (2018). Honokiol protects pancreatic *β* cell against high glucose and intermittent hypoxia-induced injury by activating Nrf2/ARE pathway in vitro and in vivo. *Biomedicine & Pharmacotherapy*.

[50] Yagishita Y., Fukutomi T., Sugawara A. (2014). Nrf2 protects pancreatic *β*-cells from oxidative and nitrosative stress in diabetic model mice. *Diabetes*.

