# Supplementary material for: Nrf2 Activation Protects Mouse Beta Cells from Glucolipotoxicity by Restoring Mitochondrial Function and Physiological Redox Balance
Source: Oxid Med Cell Longev. 2019 Nov 11;2019:7518510. doi: 10.1155/2019/7518510 (PMC6885177; doi:10.1155/2019/7518510)
Supplement: Supplementary Materials — Comparison of the effect of substimulatory glucose concentrations on acute changes in ROS balance. Islet cells were acutely treated with 0.5, 3, or 15 mmol/L glucose. Levels of DHEox (A) and DCF (B) fluorescence were similar with the two substimulatory glucose concentrations but change in response to 15 mmol/L glucose. Numbers in bars indicate the number of cells. ∗∗∗p ≤ 0.001. [file 7518510.f1.pdf]

Supplementary Figure 1

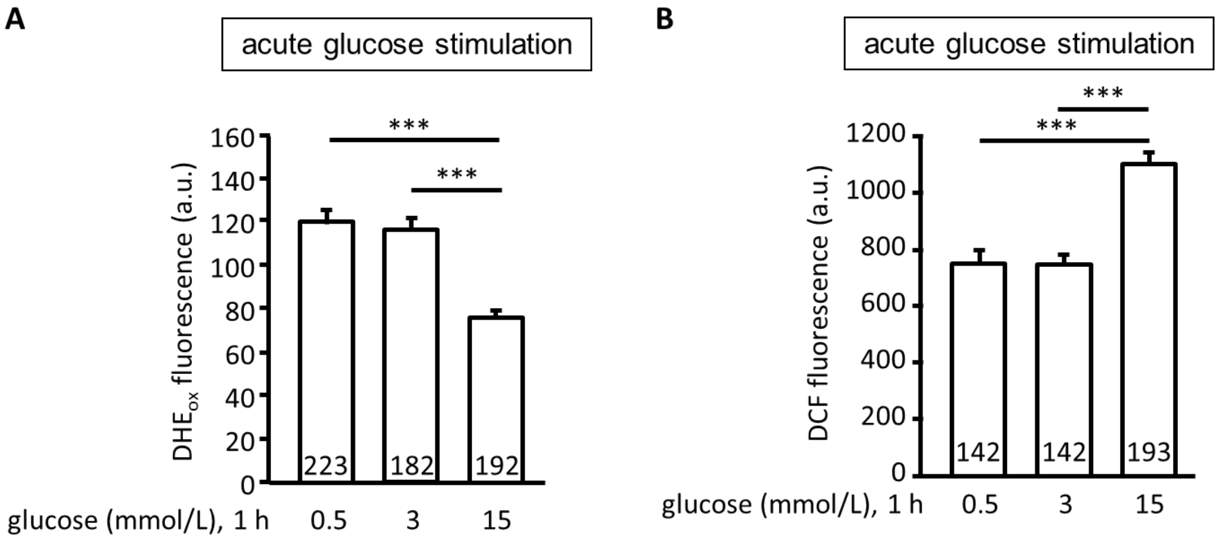

Comparison of the effect of sub-stimulatory glucose concentrations on acute changes in ROS balance.

Islet cells were acutely treated with 0.5, 3 or 15 mmol/L glucose. Levels of DHE_{ox} (A) and DCF (B) fluorescence were similar with the two sub-stimulatory glucose concentrations but change in response to 15 mmol/L glucose. Numbers in bars indicate the number of cells. ***$p \leq 0.001$
